# Comparing Tourniquet Use and Non-Use in Robot-Assisted Total Knee Arthroplasties

**DOI:** 10.3390/medicina61091701

**Published:** 2025-09-18

**Authors:** Keun Young Choi, Man Soo Kim, Yong In

**Affiliations:** Department of Orthopaedic Surgery, Seoul St. Mary’s Hospital, College of Medicine, The Catholic University of Korea, Seoul 06591, Republic of Korea; heaxagon@hanmail.net (K.Y.C.); kms3779@naver.com (M.S.K.)

**Keywords:** tourniquet, robot-assisted TKA, estimated blood loss

## Abstract

*Background and Objectives*: Performance of robot-assisted total knee arthroplasty (TKA) procedures has continued to increase in popularity. However, tourniquet use is necessary for longer periods of time in robot-assisted TKA than conventional manual TKA because the robot-assisted procedure requires an additional registration process. The use of tourniquets for long periods increases the risk of hidden blood loss and ischemic soft tissue injury in the lower extremity. The purpose of this study was to compare the value of performing robot-assisted TKA without the use of a tourniquet to that of performing this surgery with the use of a tourniquet. Parameters we assessed were blood loss, degree of postoperative thigh and knee pain, and occurrence of early post-operative complications. *Materials and Methods*: Data from 100 consecutive patients who underwent primary unilateral robot-assisted TKA between July 2024 and July 2025 were included in this study’s analyses. Patients were divided into three groups chronologically. The first 29 patients comprised group 1, the early tourniquet group; the next 30 patients were assigned to group 2, the no tourniquet group; and group 3 was the late tourniquet group and comprised the remaining 41 subjects. However, because allocation was chronological rather than randomized, the outcomes of later groups may partly reflect the surgeon’s accumulated experience (learning curve), which should be considered when interpreting the results. The primary outcome measure was estimated blood loss (EBL). The secondary outcome measures included transfusion rate, visual analog scale (VAS) pain scores for the knee and thigh on the third postoperative day, readmission rate due to surgical complications, superficial and deep infection rate, length of operation, and length of tourniquet use. *Results*: Group 2 participants, the no tourniquet participants, experienced significantly greater EBL on postoperative days (PODs) 1, 2, and 3 compared to the subjects assigned to groups 1 and 3 (*p* = 0.003, *p* < 0.001, and *p* = 0.005, respectively). However, there were no significant differences in transfusion rates (*p* = 0.290) among the 3 groups. VAS scores for knee and thigh pain were also not significantly different among the three groups (all *p*-values > 0.05). Three patients in group 1 (10.3%), one patient in group 2 (3.3%), and one patient in group 3 (2.4%) were readmitted for complications related to wound healing (*p* = 0.289). Additionally, two patients in group 1 developed superficial wound infections from which the causative bacteria were cultured. No infections were observed in the other groups (*p* = 0.082), however. Two patients in group 1 and two patients in group 2 experienced symptomatic deep vein thrombosis (DVT) (*p* = 0.235). No group 3 patients experienced DVT, and only one patient in group 2 was confirmed with DVT using an enhanced CT scan (*p* = 0.308). Group 3 patients had shorter lengths of surgery (*p* < 0.001) than group 1 and 2 patients and had shorter periods of tourniquet use (*p* = 0.034) than group 1 patients. *Conclusions*: Tourniquet non-use in robot-assisted TKA surgeries was associated with greater EBL in acute postoperative periods, but this finding was not accompanied by any change in transfusion rate. Tourniquet non-use was not clinically beneficial for reducing immediate postoperative thigh and knee pain or reducing the prevalence of early post-operative complications. Tourniquet use in robot-assisted TKA may be beneficial because of the advantages its use provides in maintaining a clear surgical field and in facilitating the cementing process.

## 1. Introduction

Performance of total knee arthroplasties (TKAs) is now the customary preferred treatment for advanced knee osteoarthritis (OA) [1]. Recently various TKA procedural alignment concepts have been introduced to improve patient satisfaction [2]. Robot-assisted TKA has been implemented to assist in the planning and execution of these surgeries. Robotic assistance in these procedures improves surgical precision and facilitates more consistently favorable TKA surgical outcomes [3]. To implement individualized alignment strategies accurately, the performance of robot-assisted TKA has become increasingly popular in clinical practice [4,5]. Robot-assisted TKA was implemented in 0.35% of 2010 cases in the United States. This percentage peaked at 4.39% in 2019 and plateaued at 3.45% in 2022 [6].

Robot-assisted TKA surgical procedures have several advantages when compared to conventional manual TKA procedures, particularly in effecting accurate component positioning, minimizing blood loss, and reducing surgical errors [1]. A disadvantage, however, is that robot-assisted TKAs require longer operation times than manual TKAs [1,5,7,8]. This robot-assisted operation is especially extended during the surgeon training period because of the learning curve associated with the procedure [1,9]. Tourniquets are usually used during robot-assisted TKAs even though their use requires additional surgery time [1,10], and their prolonged use has been associated with several complications. These complications include hidden blood loss, occurrence of ischemic soft tissue injury, increased incidence of postoperative pain, increased risk of postoperative infection and thromboembolic events, and rehabilitation delay [1,11,12]. To avoid encountering these potential disadvantages, some surgeons have attempted to perform TKAs without the use of tourniquets. The benefits and risks associated with these attempts have not, however, convincingly demonstrated value in not using tourniquets [13,14,15]. In this study, we sought to test the hypothesis that robotic-assisted TKA performance without the use of tourniquets has advantages over performance of this procedure using tourniquets. The parameters assessed in determining these potential benefits included blood loss and transfusion rate, postoperative thigh and knee pain and complication rate reduction, and variables associated with performance of the surgery. Therefore, we specifically extended our hypothesis statement to “tourniquet non-use during robot-assisted TKA has no effect on estimated blood loss (EBL), and decreases occurrence of postoperative pain, and prevalence of surgical complications in the early postoperative period.”

## 2. Materials and Methods

Between July 2024 and July 2025, 121 patients underwent primary unilateral robot-assisted TKAs using the Robotic Arm Interactive Orthopedic System (RIO) (MAKO, Stryker, Portage, MI, USA). From our analyses, we excluded data from patients with a history of prior ipsilateral knee surgery affecting bony structures, those with chronic liver disease associated with elevated serum aspartate aminotransferase and alanine aminotransferase levels more than three times the normal values, those treated for anemia within the month prior to surgery, and those with flexion contracture > 25 degrees indicating severe limitation of motion. Additionally, for the patients using an anticoagulant agent or antiplatelet agents, proper medical department consultation was done. Patients who could not cease taking those medications or needed heparin bridging were excluded. Patients who could temporarily cease anticoagulants or antiplatelet agents were recommended to stop taking those medications according to medical department recommendations and resumed taking medications after postoperative day (POD) 2 in accordance with the recommendations of the appropriate specialists. Data from twenty-one patients were excluded from the analyses; data from the remaining 100 patients were, therefore, included in this study. Patients were divided into three groups by chronology. The first 29 patients comprised group 1, the early tourniquet group; the next 30 comprised the no tourniquet group, group 2; and the late tourniquet group, group 3, included the remaining 41 patients. This retrospective case–control study was approved by the Institutional Review Board of our hospital (KC25RISI0565). This research is approved by the Seoul St. Mary’s Hospital Data Review Board.

All surgeries were performed under general anesthesia by one of this manuscript’s authors, the senior surgeon, using cemented cruciate-retaining (CR) or posterior-stabilized (PS) prostheses (Triathlon; Stryker, Portage, MI, USA). The senior surgeon has more than 20 years of experience in performance of the conventional manual TKA and mastered the learning curve associated with performance of robot-assisted TKAs. All procedures were executed using a subvastus approach with patellar sliding. For the group in whom tourniquets were used, these tourniquets were inflated before skin incision and were deflated after cement hardening. For the patients in the no tourniquet group, no tourniquet inflation was implemented at any time during TKA performance. The femoral and tibial prostheses were fixed with two packages of bone cement through a vacuum mixing system and a one-stage cementation method. The patellae were not resurfaced in any of the 100 cases. Following the routine robot-assisted TKA procedure, meticulous care was employed to ensure maintenance of hemostasis, and no drainage was established in any of the patients. Capsular and subcutaneous layers were closed followed by skin closure using a 2-octyl cyanoacrylate adhesive (Dermabond, Ethicon, Bridgewater, NJ, USA) and a self-adhesive polyester mesh (Prineo, Ethicon, Bridgewater, NJ, USA). No local infiltration anesthesia was administered to any of the 100 patients, and no nerve blocks were performed.

A consistent perioperative management protocol was followed in all cases. Post-operation, on the day of surgery, patients were instructed to extend their knees fully in sitting position. Ambulation using a walker and active attainment of maximum range of motion was encouraged on POD 1. Patient follow-up visits occurred at two and six weeks and at three and six months post-surgery. Follow-up assessments were also conducted one year post-surgery and once yearly thereafter.

Contemporary perioperative blood management (PBM) protocols were extended to all patients with the administration of systemic, intravenous tranexamic acid (TXA), 500 mg, in normal saline, 100 mL, once an hour after the skin incision was initiated. Finally, the transfusion threshold was set at a hemoglobin level < 7.0 g/dL within 3 days after surgery. However, for patients with symptoms of anemia, this transfusion threshold was 8.0 g/dL. Antithrombotic treatment was initiated on the first POD; all 100 patients received 0.2 mL, 2000 IU, subcutaneous enoxaparin (Clexane, Sanofi-Aventis, France) for five days at 24 h intervals. No patients required joint aspiration for postoperative hemarthrosis even though drainage was not established.

The primary outcome measure was EBL as calculated using the Lopez-Picado formula [16]:

$$\frac{\text{Patient}\;\text{blood}\;\text{volume}\;(\text{PBV})\times (\text{preoperative}\;\text{Hct}-\text{postoperative}\;\text{day}\;1\;\text{or}\;\text{day}\;2\;\text{or}\;\text{day}\;3\;\text{Hct})}{\text{mean}\;\text{Hct}\;\text{during}\;\text{this}\;\text{time}\;\text{frame}}$$

Patient blood volume (PBV) was calculated using the International Council for Standardization in Hematology (ICSH) formula [17]. Secondary outcome measures included blood metabolism variables including transfusion rates, hemoglobin (Hb) and hematocrit (Hct) levels; patient-reported outcome measures (PROMs) including postoperative thigh pain and knee pain evaluated using the visual analogue scale (VAS); clinical outcomes including occurrence of wound complications or superficial and deep infections, occurrence of deep vein thrombosis (DVT) symptoms, and occurrence of CT-confirmed DVTs; and surgical variables including operation time, tourniquet time, and implant types used, CR versus PS.

Subgroup analysis was performed to test for an EBL difference between the total tourniquet group (group 1 plus group 3 participant data) and no tourniquet group (group 2 data). Additionally, tourniquet time between group 1 and group 3 subjects was compared.

### Statistical Analysis

In comparing primary and secondary outcomes, we used one-way analysis of variance (ANOVA) and chi-squared tests for continuous and categorical variable analysis, respectively. Additionally, a Bonferroni correction was applied for post hoc comparisons. A power analysis indicated that 80% power was required to detect a 250 mL difference in the amount of total blood loss through two-sided testing at a significance level of 0.05. Statistical analyses were performed using SPSS version 28 (IBM Corp, Armonk, NY, USA), and statistical significance was set at *p* < 0.05.

## 3. Results

There were no significant demographic differences in age, height, weight, sex, and side of the surgical site among the three patient groups (Table 1). Group 2 participants demonstrated significantly greater EBL on PODs 1, 2, and 3 compared to groups 1 and 3 (*p* = 0.003, *p* < 0.001, and *p* = 0.005, respectively) (Table 2). Although preoperative Hb and Hct levels were not different among the groups, Hb and Hct at POD 2 were significantly different (*p* = 0.010) (Table 2). Post hoc analysis demonstrated that the Hb and Hct levels on POD 2 were significantly higher in group 3 patients than both in group 1 and group 2 subjects. These levels in group 1 and group 2 participants did not differ significantly. Also, there were no significant differences in transfusion rates (*p* = 0.290) (Table 2).

VAS scores for knee and thigh pain were not significantly different among the groups (Table 3), and the requirement for hospital readmission due to surgical complications also did not differ significantly among the groups (*p* = 0.289). Three patients in group 1, one patient in group 2, and one patient in group 3 were readmitted for surgical site issues (Table 4). Additionally, infection rates were not significantly different among the groups; two patients in group 1 developed superficial bacterial infections resulting in positive cultures of surgical site swabs (*p* = 0.082) (Table 4). Comparing symptoms suggesting DVT, patients demonstrated no significant differences among the groups (*p* = 0.235). Also, CT-confirmed DVT case numbers did not differ significantly. Only one patient in group 2 was confirmed with DVT on an enhanced CT scan (*p* = 0.308) (Table 4).

Operation times did, however, differ significantly among the groups (*p* < 0.001) (Table 5). Post hoc analysis demonstrated that the operation time for group 3 patients was significantly shorter than for both group 1 and group 2 patients, and the time for group 1 and group 2 patients did not differ. In addition, group 3 patients (48.9 ± 4.8 min) demonstrated a significantly shorter tourniquet time compared to group 1 subjects (56.4 ± 17.2 min) (*p* < 0.034). Although femoral size, tibial size, and insert thickness showed no statistically significant differences, the proportion of CR-type implants was significantly higher in group 3 compared to group 1 patients (85.4% vs. 51.7%, *p* = 0.006).

In subgroup analysis, the total tourniquet group (group 1 plus group 3 patients) demonstrated less EBL at PODs 1, 2, and 3 than the no tourniquet group (group 2) patients (tourniquet group: 398.8 ± 123.0 mL, 564.0 ± 172.5 mL, and 638.5 ± 177.7 mL, versus no-tourniquet group: 500.1 ± 151.8 mL, 723.2 ± 194.4 mL, and 781.9 ± 212.9 mL, *p* = 0.001, *p* < 0.001, and *p* < 0.001, respectively) without difference in operation time (tourniquet group: 90.8 ± 14.5 min versus no tourniquet group: 96.4 ± 11.1 min, *p* = 0.063).

## 4. Discussion

The most important finding of this study was that the group 2, no tourniquet group, subjects demonstrated significantly greater EBL among the three groups during the early postoperative period without any differences in transfusion rate. Additionally, the no tourniquet group patients (*n* = 30) had significantly greater EBL than the total tourniquet group (group 1 plus group 3 patients, *n* = 70) on subgroup analysis.

Tourniquet use in TKA procedures has been accepted as a safe and surgeon-friendly method for control of bleeding without an increase in early postoperative complications [18]. Although two studies reported negative effects of tourniquet use in TKA [1,11], most surgeons continue to use tourniquets to achieve better surgical field visualization, decrease intraoperative blood loss, and improve cementation of implants during TKAs [10,18,19]. However, the results of advantage versus disadvantage assessments of using tourniquets in primary TKAs vary, especially in cementless TKAs, a recently re-introduced surgical technique [13]. Generally, robot-assisted TKAs are associated with longer operation times than manual TKAs [1,5,7,8,9] and may cause higher intraoperative blood losses, increased soft tissue injuries, or higher complication rates due to tourniquet use [1,10]. Therefore, we tried to determine whether no use of tourniquet in robot-assisted TKA could have clinical benefits in the performance of robot-assisted TKAs.

TKA performance is occasionally associated with excessive bleeding, and tourniquet use may assist in ensuring a bloodless TKA visual surgical field and in preventing acute postoperative bleeding [20]. Tourniquets reduce intraoperative, but not total, blood loss [21], but their effects on transfusion rates, range of motion outcomes, and thromboembolic risk have not been firmly established and are subjects of debate [1,11,13,22]. Huang et al. reported that the use of a tourniquet during TKA significantly increases the total blood loss and does not decrease the post-operative transfusion rate and that using a tourniquet in performance of routine TKAs exacerbates the early post-operative hypercoagulable status and results in a higher incidence of below-knee asymptomatic DVT [11]. Omer et al. reported that the use of a tourniquet did not result in significant differences in outcomes up to three months postoperatively. The outcomes assessed included isokinetic muscle strength, pain, range of motion, and Knee Society Score (KSS) [22]. However, Lai et al. reported that in robot-assisted TKA, intraoperative bleeding was reduced in the tourniquet group, but hidden blood loss was greater. This resulted in the finding of no significant difference in total blood loss between the two groups [1]. While that study found no difference in the incidence of thromboembolic events between the groups, those researchers reported that tourniquet use in robot-assisted TKA was associated with increased postoperative pain and prolonged postoperative recovery as well as aggravation of muscle injury. The small sample size, 14 patients in each group, in that study, however, dictates that further testing is required to validate the results. Additionally, the inconsistency of their findings with previous study results may also have been caused by heterogeneity of blood management protocols and surgical procedures. Therefore, clinical data for comparing the effectiveness of tourniquet use and non-use in robot-assisted TKAs is lacking.

In this study, we identified no significant transfusion rate differences among the three groups. Although Hb and Hct levels at POD 2 were significantly higher in group 3, the late tourniquet group, the differences were not substantial enough to affect transfusion rate (Table 2). These results are consistent with those of previous studies [1,13] that also reported no difference in transfusion rate. Therefore, our results indicate that while effective in reducing EBL, tourniquet use did not have a clinical effect on transfusion rate. Recent study reports, however, suggest that following unilateral TKA, transfusion rate is markedly decreased. Our finding, therefore, may be more attributable to the presence of a significantly lower baseline transfusion rate than to a tourniquet use effect. The lower baseline transfusion rate is the product of the adoption of modern perioperative blood management protocols [23].

Our study indicated that tourniquet application did not affect clinical outcomes such as the degree of acute postoperative knee or thigh pain; because we did not perform local infiltration anesthesia or nerve blocks after surgery in any of the study’s patients, the effect of such pain control methods was negligible. These results are consistent with those of a previous study that reported no improvements in thigh muscle strength, range of motion, KSS score, and postoperative pain in the absence of tourniquet use [22]. This paradoxically suggests that the additional pain caused by tourniquet application does not significantly affect the amount of overall pain experienced by patients undergoing TKA, a finding that is probably due to the substantial postoperative pain inherently associated with the procedure [24]. Although pain management and its assessment after TKA may vary in timing and method, our findings suggest that the use of a tourniquet in robot-assisted TKA does not result in clinically significant patient self-reported pain.

Our analysis does not support the hypothesis that applying a tourniquet has a measurable effect on the rate of complications. There were no significant differences among the three groups in the readmission rate arising from surgical and overall complications, infections, necessity of DVT evaluations, and enhanced CT scan-confirmed DVT cases. Our results concur with those of a previous randomized controlled study in which the use of an optimized tourniquet did not increase the incidence of primary TKA postoperative complications [25]. However, our results do not agree with those of another previous study in which higher incidences of wound problems, leg swelling, and DVTs were reported [26]. These previous results were, however, based on performance of conventional, not robot-assisted TKA procedures, and the conflicting findings highlight the need for further randomized controlled trials with larger cohorts that specifically target patients undergoing robot-assisted TKA. Nonetheless, based on our results, the appropriate use of a tourniquet in robot-assisted TKA may offer advantages such as facilitating a clearer surgical field and minimizing interference during the cementation process. These identified advantages would enable improved surgical performance.

Operation times were significantly different among our three groups. Group 3, the late tourniquet group, operation times were significantly shorter than those of both group 1 and group 2, but no significant difference in operation times was observed between group 1 and group 2. In addition, group 3 demonstrated a significantly shorter tourniquet application time compared to group 1. These findings demonstrated that increased experience of the surgeon and the surgeon’s mastery of the initial learning curve result in reductions in both operative and tourniquet application times. This result coincides with those of a previous study reporting that shorter average operating times are associated with every 30 cases of early surgical experience [27]. The mastery of the learning curve in robot-assisted TKA is associated with a reduction in tourniquet time, contrary to concerns raised in recent reports; these lengths eventually equilibrate to those observed in the performance of conventional TKAs [28,29]. Despite the reduction in surgical time, our group 2, the no tourniquet group, demonstrated a significantly higher EBL than the other groups. Therefore, these results imply that in robot-assisted TKA, reduced operative time alone does not necessarily translate to lower blood loss; however, the use of a tourniquet may contribute substantially to minimizing actual blood loss.

This study had several limitations. First, patient grouping was performed retrospectively rather than through prospective randomization, and group allocation was performed based on chronology. Results may, therefore, have been influenced by the evolution of surgical prowess. In particular, the outcomes of the later groups may partly reflect the surgeon’s accumulated experience (learning curve), introducing potential bias. The early tourniquet group included patients who underwent surgery with tourniquet use after the surgeon’s mastery of the initial learning curve; the no tourniquet group consisted of patients treated without a tourniquet based on the assumption that tourniquet non-use might be beneficial for robotic-assisted TKAs, a procedure that requires more time operative than conventional TKAs; and the late tourniquet group included patients in whom the tourniquet was reintroduced into the procedure due to issues of excessive bleeding resulting in poor visualization of the surgical field and difficulties with the cementation process. Performance of a prospective randomized study with more patients is warranted to compare the effects of tourniquet use or non-use in robot-assisted TKA more thoroughly. Second, the generalizability of our findings may be limited. Our study population was confined to Koreans, and most of the patients included in this study were women (72/100, 72%). The reason for this increased propensity of Korean women to develop arthritis has not been elucidated [30]. Third, the statistical power resulting from our sample size calculations may have been less than adequate for detecting all relevant outcomes. Type II errors associated with detection of these outcomes may have been present. Fourth, perioperative blood management and postoperative rehabilitation protocol heterogeneity between institutions and surgeons may have affected study result comparisons. However, the protocols applied to all 100 patients in this study were consistent. Fifth, our patient wound and complication status follow-up period was short, one month. The lack of generalizability of our findings due to the identified limitations needs to be emphasized, but, despite these limitations, we have provided valuable information on blood loss, clinical outcomes, and surgical consideration effects of tourniquet non-use in robot-assisted TKAs.

## 5. Conclusions

Tourniquet non-use in robot-assisted TKA was associated with greater EBL in the acute postoperative period without affecting transfusion rate. Tourniquet non-use did not provide clinical benefits in reducing immediate postoperative pain or complications. Because group allocation was chronological, outcomes may have been influenced by the surgeon’s learning curve. Since the increase in surgeon’s experience was associated with robot-assisted TKA operation times approximating those of manual TKAs, we recommend the use of tourniquets in robot-assisted TKAs to allow for better visualization of the surgical field and to facilitate the cementing process.

## Figures and Tables

**Table 1:** Demographics.

	Early Tourniquet (*n* = 29)	No Tourniquet (*n* = 30)	Late Tourniquet (*n* = 41)	*p* Value
Age ^a^	71.5 ± 5.8	71.7 ± 6.5	72.5 ± 5.7	0.736
Height ^a^	1.58 ± 0.07	1.57 ± 0.09	1.58 ± 0.06	0.911
Weight ^a^	63.5 ± 6.4	64.7 ± 13.6	63.6 ± 7.3	0.862
Male sex †	5 (17.2%)	8 (26.7%)	4 (9.8%)	0.173
Female sex †	24 (82.8%)	22 (73.3%)	37 (90.2%)	
Op side Rt. †	15 (51.7%)	13 (43.3%)	15 (36.6%)	0.452
Op side Lt. †	14 (48.3%)	17 (56.7%)	26 (63.4%)	

^a^ Data are presented as means ± standard deviations. † Data are presented as numbers (percentage) of patients. Op: operation.

**Table 2:** Estimated blood loss and hemodynamic variables and post hoc analysis.

	Early Tourniquet (*n* = 29)	No Tourniquet (*n* = 30)	Late Tourniquet (*n* = 41)	*p*-Value
EBL POD 1 ^a^	407.9 ± 133.8	500.1 ± 151.8	392.3 ± 116.0	0.003
EBL POD 2 ^a^	595.6 ± 181.7	723.2 ± 194.4	542.3 ± 164.6	<0.001
EBL POD 3 ^a^	622.3 ± 180.8	781.9 ± 212.9	647.5 ± 177.6	0.005
Transfusion rate †	1 (3.4%)	0 (0.0%)	0 (0.0%)	0.290
Preop Hb ^a^	13.0 ± 1.1	13.7 ± 1.4	13.3 ± 1.1	0.106
Preop Hct ^a^	40.0 ± 3.2	41.7 ± 4.6	41.3 ± 3.3	0.149
POD1 Hb ^a^	10.5 ± 1.1	10.4 ± 1.3	10.8 ± 1.1	0.182
POD1 Hct ^a^	32.1 ± 3.2	32.1 ± 3.8	33.4 ± 3.3	0.163
POD2 Hb ^a^	9.4 ± 1.1	9.1 ± 1.2	10.0 ± 1.1	0.010
POD2 Hb ^a^*	9.4 ± 1.1	9.1 ± 1.2		1.000 *
POD2 Hb ^a^*	9.4 ± 1.1		10.0 ± 1.1	0.147 *
POD2 Hb ^a^*		9.1 ± 1.2	10.0 ± 1.1	0.011 *
POD2 Hct ^a^	29.0 ± 3.6	28.4 ± 3.5	30.8 ± 3.6	0.011
POD2 Hb ^a^*	29.0 ± 3.6	28.4 ± 3.5		1.000 *
POD2 Hb ^a^*	29.0 ± 3.6		30.8 ± 3.6	0.114 *
POD2 Hb ^a^*		28.4 ± 3.5	30.8 ± 3.6	0.014 *
POD3 Hb ^a^	92 ± 0.9	8.8 ± 1.2	9.4 ± 1.1	0.061
POD3 Hct ^a^	28.5 ± 3.1	27.4 ± 3.5	29.1 ± 3.3	0.126

^a^ Data are presented as means ± standard deviations. † Data are presented as numbers (percentage) of patients. * Values after post hoc analysis by Bonferroni correction. EBL: Estimated blood loss. Hb: Hemoglobin. Hct: Hematocrit.

**Table 3:** Surgical site pain and thigh pain.

	Early Tourniquet (*n* = 29)	No Tourniquet (*n* = 30)	Late Tourniquet (*n* = 41)	*p*-Value
Knee pain ^a^	5.0 ± 2.8	5.3 ± 1.3	5.9 ± 1.8	0.323
Thigh pain ^a^	3.5 ± 0.7	3.4 ± 2.1	4.1 ± 2.6	0.553
Radiating pain ^a^	0.0 ± 0.0	0.2 ± 0.9	0.1 ± 0.4	0.754

^a^ Data are presented as means ± standard deviations.

**Table 4:** Complications.

	Early Tourniquet (*n* = 29)	No Tourniquet (*n* = 30)	Late Tourniquet (*n* = 41)	*p*-Value
Readmission Rate	3 (10.3%)	1 (3.3%)	1 (2.4%)	0.289
Superficial Infection ^†^	2 (6.9%)	0 (0.0%)	0 (0.0%)	0.082
DVT Symptom ^†^	2 (6.9%)	2 (6.7%)	0 (0.0%)	0.235
CT-confirmed DVT ^†^	0 (0.0%)	1 (3.3%)	0 (0.0%)	0.308
Overall Complication ^†^	5 (17.2%)	3 (10.0%)	11 (26.8%)	0.195

^†^ Data are presented as numbers (percentage) of patients. CT: computed tomography. DVT: deep vein thrombosis.

**Table 5:** Surgical variables.

	Early Tourniquet (*n* = 29)	No Tourniquet (*n* = 30)	Late Tourniquet (*n* = 41)	*p*-Value
Femur Size ^a^	3.4 ± 1.0	3.7 ± 1.5	3.4 ± 1.0	0.403
Tibia Size ^a^	3.4 ± 1.0	3.8 ± 1.4	3.4 ± 1.0	0.264
Insert ^a^	11.1 ± 1.5	11.1 ± 1.7	10.9 ± 1.5	0.800
Implant Type				
CR ^†^	15 (51.7%)	23 (76.7%)	35 (85.4%)	0.007
PS ^†^	14 (48.3%)	7 (23.3%)	6 (14.6%)	
	15 (51.7%)	23 (76.7%)		0.136 *
	15 (51.7%)		35 (85.4%)	0.006 *
		23 (76.7%)	35 (85.4%)	1.000 *
Op Time ^a^	100.8 ± 15.5	96.4 ± 11.1	83.7 ± 8.3	<0.001
	100.8 ± 15.5	96.4 ± 11.1		0.431 *
	100.8 ± 15.5		83.7 ± 8.3	<0.001 *
		96.4 ± 11.1	83.7 ± 8.3	<0.001 *
Tourniquet Time ^a^	56.4 ± 17.2		48.9 ± 4.8	0.034

^a^ Data are presented as means ± standard deviations. ^†^ Data are presented as numbers (percentage) of patients. * Values after post hoc analysis by Bonferroni correction. CR: Cruciate-retaining, PS: Posterior-stabilized.

## Data Availability

Due to privacy and ethical restrictions, the data of this study are not publicly available.
